# Glycyrrhetinic Acid Receptor-Mediated Zeolitic Imidazolate Framework-8 Loaded Doxorubicin as a Nanotherapeutic System for Liver Cancer Treatment

**DOI:** 10.3390/molecules28248131

**Published:** 2023-12-16

**Authors:** Xiao Mi, Yang Lou, Yutian Wang, Mingran Dong, Hongwei Xue, Shuyang Li, Juan Lu, Xi Chen

**Affiliations:** Institute of Medicinal Plant Development, Chinese Academy of Medical Sciences, Peking Union Medical College, Beijing 100193, China; mixiao.tsin@foxmail.com (X.M.); louyang0727@163.com (Y.L.); wytqingya@163.com (Y.W.); dongmingran@implad.ac.cn (M.D.); 15297318065@163.com (H.X.); 15563999708@163.com (S.L.)

**Keywords:** ZIF-8, glycyrrhetinic acid, DOX, nanotherapeutic system, liver cancer

## Abstract

In this study, we designed and developed a DOX nanodrug delivery system (PEG-GA@ZIF-8@DOX) using ZIF-8 as the carrier and glycyrrhetinic acid (GA) as the targeting ligand. We confirmed that DOX was loaded and PEG-GA was successfully modified on the surface of the nanoparticles. The in vitro release profile of the system was investigated at pH 5.0 and 7.4. The cellular uptake, in vitro cytotoxicity, and lysosomal escape characteristics were examined using HepG2 cells. We established an H22 tumor-bearing mouse model and evaluated the in vivo antitumor activity. The results showed that the system had a uniform nanomorphology. The drug loading capacity was 11.22 ± 0.87%. In acidic conditions (pH 5.0), the final release rate of DOX was 57.73%, while at pH 7.4, it was 25.12%. GA-mediated targeting facilitated the uptake of DOX by the HepG2 cells. PEG-GA@ZIF-8@DOX could escape from the lysosomes and release the drug in the cytoplasm, thus exerting its antitumor effect. When the in vivo efficacy was analyzed, we found that the tumor inhibition rate of PEG-GA@ZIF-8@DOX was 67.64%; it also alleviated the loss of the body weight of the treated mice. This drug delivery system significantly enhanced the antitumor effect of doxorubicin in vitro and in vivo, while mitigating its toxic side effects.

## 1. Introduction

Liver cancer ranks among the most prevalent cancers worldwide and stands as a major contributor to cancer-related fatalities [1,2]. Over the last few decades, its incidence has been steadily rising in many nations [3,4]. Presently, chemotherapy remains the most effective therapeutic approach [5]. However, it faces several limitations, including poor precision in drug delivery, rapid drug breakdown, and severe adverse reactions [6]. Consequently, targeted chemotherapy emerges as a more promising alternative, offering the potential to minimize the toxic side effects and enhance the treatment effectiveness by directing drugs specifically to the cancer cells [7]. GA, a bioactive metabolite of glycyrrheic acid derived from the root of licorice, can bind to glycyrrhetinic acid receptors (GA-R) on the surface of hepatic parenchymal cells, allowing it to enter these cells and exert its liver-targeting effect [2,8,9]. As a result, integrating GA into a nano drug delivery system (NDDS) can enhance the drug uptake by liver tumor cells [10,11].

Doxorubicin (DOX) stands as one of the most widely employed and effective broad-spectrum anticancer chemotherapy drugs in clinical practice. It is primarily used in the treatment of various tumors, including acute leukemia [12,13], malignant lymphoma [14], breast cancer [15,16], lung cancer [17], and liver cancer [18,19,20]. Nevertheless, its lack of specificity affects all the cell types in the body, leading to severe adverse reactions and constraining its clinical utility [2,21]. To enhance DOX’s antitumor activity and diminish its toxic side effects, a targeted DOX delivery system presents an effective solution [22,23,24,25].

Currently, a myriad of materials have been developed as carriers for NDDS, driving significant advancements in the realms of tumor treatment, imaging, and diagnosis [26,27,28]. In contrast to organic nanocarriers with low drug loading capacity and stability, as well as inorganic nanomaterials with restricted biocompatibility and degradability, organic–inorganic hybrid nanocarriers typified by metal–organic frameworks (MOFs) adeptly merge the strengths of inorganic nanomaterials with the excellent compatibility of organic nanomaterials, thus striking a balance between the advantages and disadvantages in drug delivery platforms [29,30]. A zinc-based zeolitic imidazolate framework (ZIF) has garnered increasing attention as a drug nanocarrier due to its outstanding pH-responsive properties and high biocompatibility [31,32]. Notably, ZIF-8 has recently become a focal point of research in the field of nanomedicine [33,34,35,36,37].

Therefore, we designed a PEG-GA@ZIF-8@DOX NDDS employing ZIF-8 as the carrier and GA as the targeting ligand, with the objective of enhancing the efficacy of doxorubicin (DOX), while reducing its toxicity.

## 2. Results

### 2.1. Synthesis and Characterization of PEG-GA@ZIF-8@DOX

**Figure 1:**
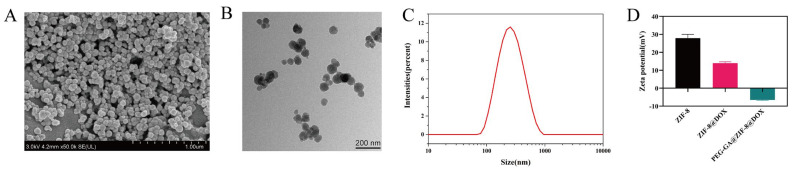
(**A**) SEM image and (**B**) TEM image of PEG-GA@ZIF-8@DOX. (**C**) Particle size distribution of PEG-GA@ZIF-8@DOX. (**D**) Zeta potential of ZIF-8, ZIF-8@DOX and PEG-GA@ZIF-8@DOX.

The PEG-GA@ZIF-8@DOX nano-delivery system designed to target the GA receptor was synthesized using an in situ “one-pot stirring” method [38]. As illustrated in Figure 1A,B, the SEM and TEM reveal the relatively consistent particle size of PEG-GA@ZIF-8@DOX. The hydrodynamic diameter of PEG-GA@ZIF-8@DOX, determined using a Malvern particle size analyzer (Figure 1C), measured at 236.37 ± 0.21 nm (with a PDI of 0.169 ± 0.038). Furthermore, the Zeta potential of PEG-GA@ZIF-8@DOX was found to be −6.52 ± 0.29 mV (Figure 1D).

**Figure 2:**
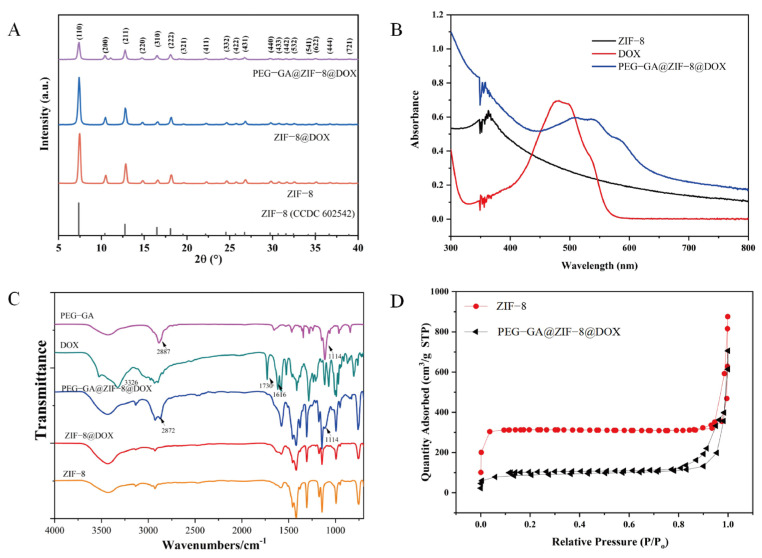
(**A**) XRD patterns of ZIF-8, ZIF-8@DOX and PEG-GA@ZIF-8@DOX. (**B**) UV absorption spectra of ZIF-8, DOX and PEG-GA@ZIF-8@DOX. (**C**) Infrared spectra of ZIF-8, ZIF-8@DOX, PEG-GA@ZIF-8@DOX, DOX, and PEG-GA were prepared. (**D**) N_2_ absorption desorption isotherms of prepared ZIF-8 and PEG-GA@ZIF-8@DOX.

The crystal structures of both ZIF-8 and PEG-GA@ZIF-8@DOX were analyzed using XRD (Figure 2A). The XRD pattern of PEG-GA@ZIF-8@DOX exhibited a similar crystal structure to that of ZIF-8, suggesting that the incorporation of DOX did not disrupt the integrity of the ZIF-8 structure. However, the surface modification of PEG-GA on the nanoparticles partially obscured the crystal structure. To confirm the encapsulation of DOX within the nanomaterial, UV-Vis spectroscopy was employed (Figure 2B). It revealed that the characteristic absorption peak of DOX at 480 nm was masked in the spectrum of PEG-GA@ZIF-8@DOX, indicating the successful encapsulation of DOX. Furthermore, Fourier-transform infrared (FT-IR) spectroscopy (Figure 2C) was utilized to verify the loading process of DOX onto the nanoplatform. A comparison of the spectra of DOX and ZIF-8@DOX showed the absence of characteristic peaks at 3326 cm^−1^ (-OH), 1730 cm^−1^ (-NH_2_), and 1616 cm^−1^ (C=O) from the DOX spectrum. This disappearance indicated that the characteristic peaks of DOX were concealed due to the physical shielding effect of ZIF-8, further confirming the successful synthesis of ZIF-8@DOX. In the spectrum of PEG-GA@ZIF-8@DOX, the appearance of characteristic peaks of PEG-GA at 2872 cm^−1^ (-CH_2_) and 1114 cm^−1^ (C-O-C) confirmed the successful modification of PEG-GA onto the ZIF-8@DOX nanoparticles. Additionally, as a control, the Brunauer–Emmett–Teller (BET) method was employed to assess the specific surface area of PEG-GA@ZIF-8@DOX nanostructures using ZIF-8 as a reference (Figure 2D). The BET specific surface area of ZIF-8 was measured at 963.59 m^2^/g, while that of PEG-GA@ZIF-8@DOX was 282.24 m^2^/g. This significant decrease in the specific surface area indicated the successful loading of DOX.

### 2.2. Drug Loading and In Vitro Release Study

**Figure 3:**
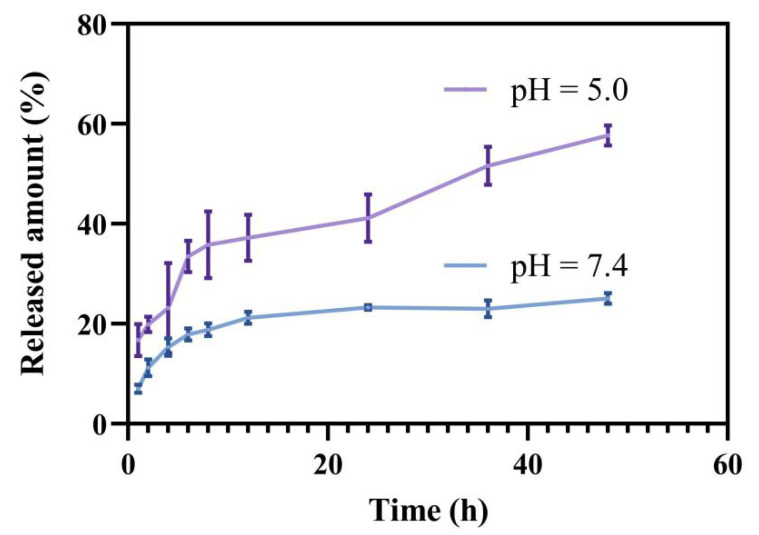
The release curve of DOX in PBS buffer solution with pH = 5.0 or pH = 7.4.

The drug loading of PEG-GA@ZIF-8@DOX was found to be 11.22 ± 0.87%, as determined from the standard curve. To explore the release of DOX, a membrane dialysis method was employed under distinct pH conditions (pH = 5.0 or pH = 7.4). As depicted in Figure 3, when placed in a PBS solution mimicking a normal physiological pH, the drug release rate at 48 h was 25.12%. Conversely, in a PBS solution mimicking the acidic environment of tumor cells, the ultimate drug release rate reached 57.73%. This highlights the acid-responsive release properties of PEG-GA@ZIF-8@DOX, suggesting its potential as a promising platform for delivering drugs to tumor sites. The limited release under acidic conditions may be due to the formation of Zn(II)-DOX coordination bonds [38].

### 2.3. Cell Toxicity Assay

**Figure 4:**
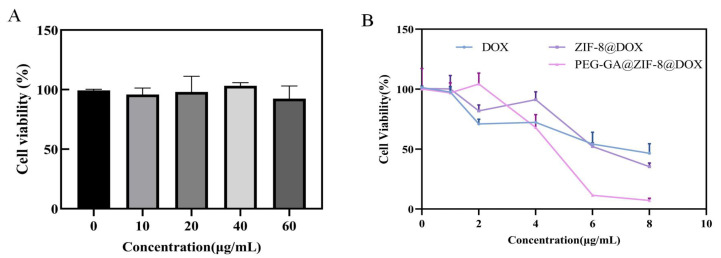
(**A**) In vitro cytotoxicity of blank nanocarriers PEG-GA@ZIF-8. (**B**) In vitro cytotoxicity of DOX, ZIF-8@DOX and PEG-GA@ZIF-8@DOX.

To assess the impact of PEG-GA@ZIF-8@DOX on the viability of HepG2 cells, the CCK-8 was utilized method to measure cell viability in response to the blank carrier and different doses of DOX, ZIF-8@DOX, and PEG-GA@ZIF-8@DOX. The cytotoxicity of PEG-GA@ZIF-8@DOX was notably stronger at high DOX concentrations compared to DOX and ZIF-8@DOX alone. Cell viability exhibited a substantial decrease, and DOX’s cytotoxicity exceeded 85% against the HepG2 tumor cells at a concentration of 6 µg/mL, demonstrating the synergistic effect of the PEG-GA@ZIF-8@DOX drug delivery system on DOX (Figure 4B).

### 2.4. Cellular Uptake Experiments

**Figure 5:**
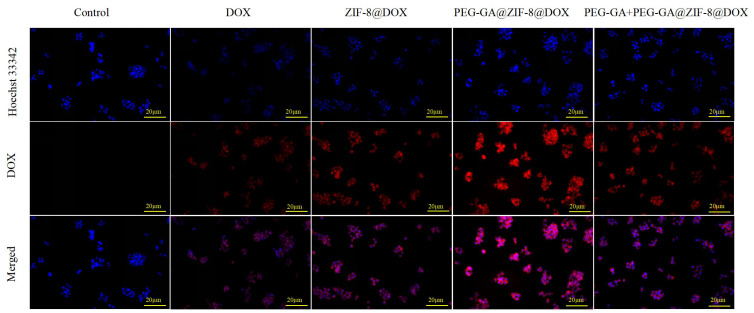
Fluorescence images of DOX, ZIF-8@DOX, PEG-GA@ZIF-8@DOX, and PEG-GA + PEG-GA@ZIF-8@DOX acting on cells for 4 h; scale bar represents 20 µm.

The process of cellular drug uptake into tumor cells is a pivotal step in achieving antitumor effects [39]. To examine the uptake of PEG-GA@ZIF-8@DOX by HepG2 cells, inverted fluorescence microscope was employed. As depicted in Figure 5, the findings reveal a substantial increase in the uptake of PEG-GA@ZIF-8@DOX in the HepG2 cells in comparison to those of DOX and ZIF-8@DOX, as evidenced by the intensified red fluorescence observed within the cells. When PEG-GA was used to compete with the GA receptors on the surface of the HepG2 cells, a reduction in the uptake of PEG-GA@ZIF-8@DOX by the HepG2 cells was observed. These outcomes suggest that the enhanced uptake of PEG-GA@ZIF-8@DOX by HepG2 cells is facilitated by the GA receptors on the cell surface.

### 2.5. Lysosomal Escape

**Figure 6:**
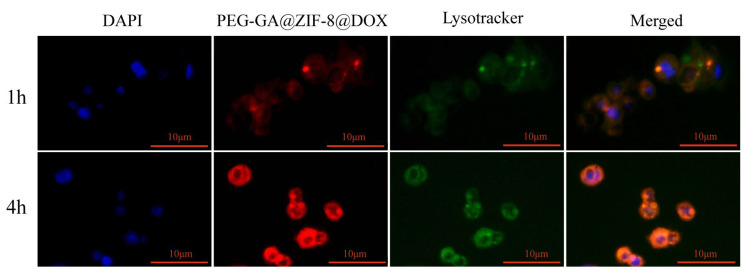
Lysosomal escape of PEG-GA@ZIF-8@DOX after co-incubation with HepG2 cells for 1 h or 4 h; scale bar represents 10 µm.

Drug molecules must traverse the cell membrane and escape the lysosomes to carry out their action within the cytoplasm. Consequently, efficient lysosomal escape is a crucial capability that drug delivery systems should possess [40]. The HepG2 cells were exposed to PEG-GA@ZIF-8@DOX for 1 h or 4 h. The cell nuclei were stained with DAPI, and the lysosomes were marked with the Lysotracker Green fluorescent probe (green). Lysosomal escape analysis was conducted using fluorescence microscopy. As illustrated in Figure 6, following the co-incubation of the HepG2 cells with PEG-GA@ZIF-8@DOX for 1 h, the red fluorescence of DOX coincided with the green fluorescence of Lysotracker, signifying that PEG-GA@ZIF-8@DOX initially entered the lysosomes upon entering the tumor cells. As the incubation time was extended to 4 h, the red fluorescence indicative of DOX increased in the cytoplasm and entered the cell nucleus. These findings affirm that PEG-GA@ZIF-8@DOX can effectively evade the lysosomes, releasing the drug into the cytoplasm, and thereby, exerting its antitumor effect.

### 2.6. Hemolytic Test

**Figure 7:**
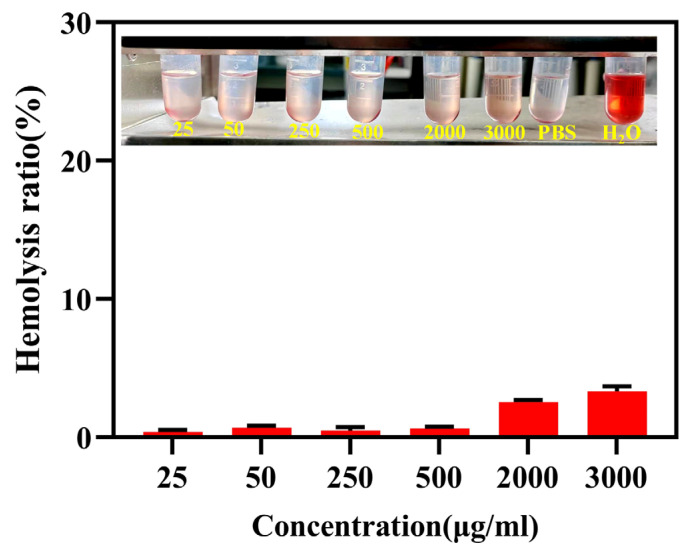
Hemolytic test of different concentrations of PEG-GA@ZIF-8@DOX.

Hemocompatibility plays a critical role in ensuring the suitability of this material for in vivo biomedical applications. To assess the hemocompatibility and biocompatibility of PEG-GA@ZIF-8@DOX, a 2% suspension of New Zealand rabbit red blood cells was employed for analysis. The hemolytic toxicity of PEG-GA@ZIF-8@DOX was tested over a concentration range of 25–3000 µg/mL, and the results are presented in Figure 7. Even at the highest concentration within the tested range, the hemolysis rate of PEG-GA@ZIF-8@DOX remained below 5%. This indicates low hemolytic toxicity of the system towards red blood cells, meeting the requirements for intravenous administration.

### 2.7. In Vivo Antitumor Activity Evaluation

**Figure 8:**
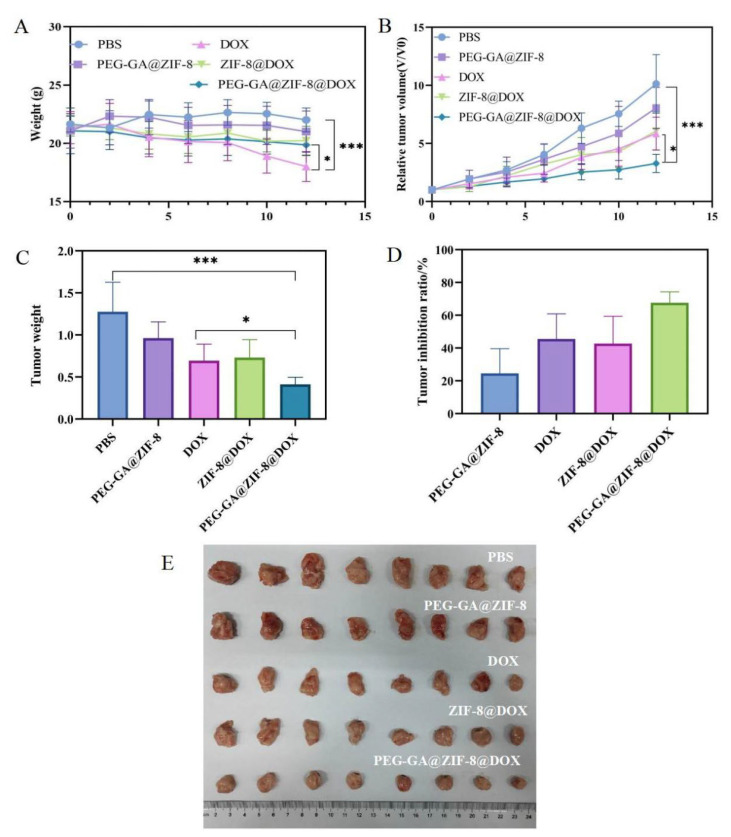
Evaluation of in vivo antitumor activity following treatment with PBS, PEG-GA@ZIF-8, DOX, ZIF-8@DOX, and PEG-GA@ZIF-8@DOX. (**A**) Changes in body weight of mice in each group. (**B**) Changes in tumor volume of mice in each group. (**C**) Tumor weight in each group. (**D**) Tumor inhibition ratio. (**E**) The final tumor morphology of mice in each group after treatment (means ± SD, n = 8). * *p* < 0.05; *** *p* < 0.001.

The in vivo pharmacological effectiveness of DOX and its nano-delivery system was assessed in the H22 hepatoma mouse model. After the administration of DOX, a noticeable decline in mouse’s body weight was observed. However, the administration of ZIF-8@DOX and PEG-GA@ZIF-8@DOX helped alleviate this decline in mouse body weight (Figure 8A). This suggests a potential reduction in DOX-induced side effects facilitated by the ZIF-8 nano-delivery system. The changes in tumor volume throughout the administration process are presented in Figure 8B. Notably, PEG-GA@ZIF-8@DOX exhibited the most favorable therapeutic effect, surpassing both the DOX and ZIF-8@DOX groups. Interestingly, no significant difference was observed between the ZIF-8@DOX and DOX groups, indicating that solely relying on the enhanced permeability and retention (EPR) effect may not be sufficient to enhance its antitumor efficacy. Figure 8C illustrates the final tumor weight, from which the tumor inhibition rates were calculated (Figure 8D). The tumor inhibition rate of the original DOX formulation was 45.52%, while the tumor inhibition rate of ZIF-8@DOX was 42.67%. Remarkably, PEG-GA@ZIF-8@DOX demonstrated a final inhibition rate of 67.64%. These results collectively highlight the enhanced therapeutic efficacy and reduced toxicity of the PEG-GA@ZIF-8@DOX nano-delivery platform in an in vivo setting. The morphological characteristics of the tumor tissue are depicted in Figure 8E. The tumor tissues in the PBS and PEG-GA@ZIF-8 groups displayed larger tumor volumes, accompanied by abundant vascularization and a darker coloration. The absence of drug intervention led to variations in the tumor morphology. In contrast, tumor growth was significantly inhibited in the DOX, ZIF-8@DOX, and PEG-GA@ZIF-8@DOX groups. Not only did the tumor volume decrease, but the number of blood vessels on the tumor surface also decreased, resulting in a lighter tumor coloration.

**Figure 9:**
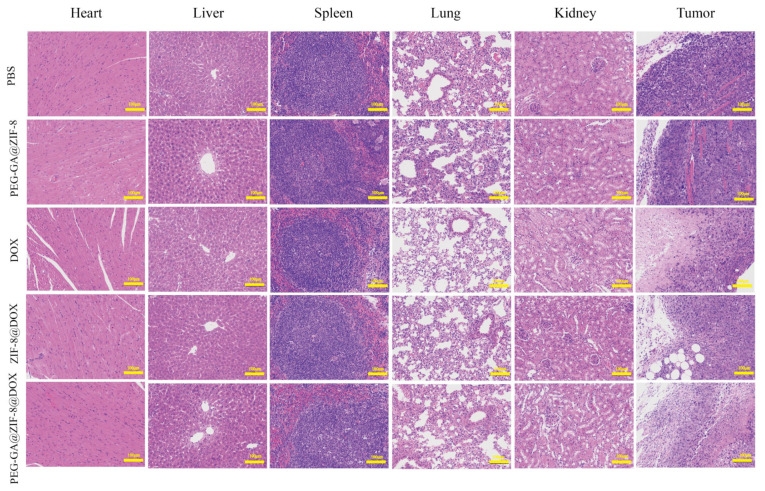
H&E staining of major organs (heart, liver, spleen, lung, and kidney) and tumor tissues in mice after PBS, PEG-GA@ZIF-8, DOX, ZIF-8@DOX and PEG-GA@ZIF-8@DOX treatment; scale bar: 100 µm.

The histopathological condition of the major organs and tumor tissues in each group was assessed using H&E staining, as depicted in Figure 9. In the tumor tissues of the PBS and blank material PEG-GA@ZIF-8 groups, we observed no significant pathological damage. Additionally, the presence of blood vessels in the tumor tissues indicated malignant tumor growth. Conversely, the tumor tissues of the DOX, ZIF-8@DOX, and PEG-GA@ZIF-8@DOX groups displayed the absence of blood vessels. We also noticed irregular changes in cell shape, varying levels of necrotic areas, nuclear condensation, and nuclear loss. Among these groups, the PEG-GA@ZIF-8@DOX group exhibited the most noticeable changes, suggesting significant damage to the tumor tissues caused by PEG-GA@ZIF-8@DOX. Cardiotoxicity is a major side effect of DOX [41], but we found no significant pathological damage in the cardiac muscle cells of the DOX group, which could be attributed to the relatively short treatment period. Moreover, the cellular integrity and histological structure of the liver, spleen, kidneys, and lungs in all the groups remained intact, indicating the good biocompatibility of PEG-GA@ZIF-8@DOX.

## 3. Discussion

Chemotherapeutic agents, such as paclitaxel (PTX), 5-fluorouracil (5-FU), and DOX, often lack selectivity between cancer cells and the neighboring normal cells, leading to significant adverse effects [9,10]. Various receptors have been identified, including the folate receptor (FA receptor) [42], GA receptor [43], the sialic acid glycoprotein receptor [44], the epidermal growth factor receptor [45], and the transferrin receptor [46]. Different tumor cells exhibit unique patterns of overexpressed surface receptors, with the GA receptors being 1.5–5 times more abundant in liver tumor tissues than they are in the neighboring normal liver tissues [47,48].

In this study, we developed a GA receptor-targeted DOX nano-delivery system, PEG-GA@ZIF-8@DOX, using ZIF-8 as the carrier and GA as the targeting molecule through an in situ “one-pot stirring” method. This system has a strong affinity for the GA-R receptors on the cell membrane of hepatic cells [49], enabling the efficient transport of the drug to the liver lesions. As a result, it enhances the drug efficacy and reduces the adverse reactions [9]. TEM and SEM images showed that PEG-GA@ZIF-8@DOX had a uniform nanoscale morphology. In comparison to ZIF-8 and ZIF-8@DOX, PEG-GA@ZIF-8@DOX had a zeta potential of −6.52 ± 0.29 mV. The research indicates that drug delivery systems with high positive charges often interact with plasma proteins, reducing their stability in the bloodstream. Conversely, systems with low negative charges enhance stability and cellular uptake [50,51]. The crystal structures of each product were investigated using XRD analysis. The results revealed that both the pristine ZIF-8 and the drug-loaded ZIF-8 solids exhibited a certain degree of crystallinity. The XRD patterns obtained were in good agreement with the reported crystal structure data of ZIF-8, further confirming the successful synthesis of ZIF-8 [52].

The results of the extracellular cellular uptake experiments confirmed that the enhanced uptake of PEG-GA@ZIF-8@DOX by the HepG2 cells was mediated through the GA-R receptors on the cell surface. In comparison to DOX and ZIF-8@DOX, PEG-GA@ZIF-8@DOX exhibited the most vibrant crimson fluorescence within the HepG2 cells, indicating a significant increase in the cellular uptake of the therapeutic agents encapsulated within PEG-GA@ZIF-8@DOX, thereby achieving targeted efficacy. Moreover, ZIF-8 exhibited pH-responsive degradation properties, enabling the release of zinc ions and encapsulated cargo under acidic conditions. Zinc ions could induce the influx of counterbalancing ions and facilitate the generation of reactive oxygen species (ROS), disrupting the integrity of intracellular/lysosomal membranes [39,53]. To protect small-molecule drugs, gene therapeutics, and proteins loaded within the lysosomes from enzymatic or acidic degradation, the ability of nanocarriers to escape the lysosomes is crucial. Once taken up by tumor cells, PEG-GA@ZIF-8@DOX achieves lysosomal escape, releasing the chemotherapy drug into the cytoplasm. This process enables the drug to effectively combat tumors. Using the H22 hepatoma mouse model, we conducted an in vivo evaluation of the antitumor activity of PEG-GA@ZIF-8@DOX. After 12 days of treatment, the tumor weight in the PEG-GA@ZIF-8@DOX group was lower than those in the groups treated with PBS, PEG-GA@ZIF-8, and DOX. This indicates that the drug delivery system has a pronounced inhibitory effect on tumors. These findings can be attributed to the increased binding specificity of PEG-GA@ZIF-8@DOX to tumors, facilitating its accumulation at the tumor site. Furthermore, the DOX treatment resulted in a significant decrease in the mice’s body weight, while PEG-GA@ZIF-8@DOX showed the potential to mitigate the decline in body weight, indicating its ability to reduce systemic toxicity. The histological analysis of tumor tissues revealed significant damage inflicted by PEG-GA@ZIF-8@DOX, demonstrating its antitumor efficacy. In future studies, the use of orthotopic liver tumor models may be considered for the in vivo evaluation of antitumor activity, as these models better simulate the tumor microenvironment of liver cancer cells, with a richer vascular network and increased blood flow [54].

## 4. Materials and Methods

### 4.1. Materials and Instruments

Hexahydrated zinc nitrate was obtained from Tianjin Fuchen Chemical Reagents Co., Ltd. (Tianjin, China), and 2-methylimidazole was acquired from TCI (Shanghai) Development Co., Ltd. (Shanghai, China). Doxorubicin Hydrochloride (with a purity of ≥99%) was received from Shanghai Winherb Medical Technology Co., Ltd. (Shanghai, China). PEG-GA was provided by Xi’an Qi Yue Biotechnology Co., Ltd. (Xi’an, China). The CCK-8 assay kit and lyso-tracker green lysosomal fluorescent probe were purchased from Shanghai Beyotime Biotechnology Co., Ltd. (Shanghai, China). The DAPI staining solution and Hoechst 33342 staining solution were received from Beijing Solabao Technology Co., Ltd. (Beijing, China). Fetal bovine serum was sourced from PAN Seratech in Germany, and 4% paraformaldehyde solution was bought from Beijing Solarbio Technology Co., Ltd. (Beijing, China).

The size and electric charge of the nanoparticles were analyzed using a Nano ZS laser particle size analyzer from Malvern Instruments Ltd. (Malvern, UK). Their shape and structure were examined with an FEI Tecnai G2 F30 transmission electron microscope (FEI, Hillsboro, OR, USA) and an SU8020 scanning electron microscope (Hitachi Ltd., Tokyo, Japan). UV-visible light absorption, drug loading, and drug release were assessed with a UV-Vis spectrophotometer (Cary100, Agilent Technologies Ltd., Santa Clara, CA, USA). The infrared spectra were determined using an infrared spectrometer (Nicolet iS 5, ThermoFisher Scientific Inc., Waltham, MA, USA). The fluorescence staining results of the in vitro cell uptake experiment and the lysosome escape experiment were observed and recorded using a fluorescence microscope (ECLIPSE Ts2R, Nikon Corporation, Tokyo, Japan).

### 4.2. Cell Lines and Animals

The human liver cancer cell line HepG2 and the H22 mouse liver cancer cell line were from the Cell Resource Center of the Institute of Basic Medical Sciences, Chinese Academy of Medical Sciences (Beijing, China). Healthy 20–22 g Balb/c mice were provided by Sibeifu (Beijing) Experimental Animal Technology Co., Ltd. (Beijing, China). All animals were kept in facilities with controlled conditions, including a temperature range of 17–25 °C, humidity maintained between 45–80%, and a 12 h light/dark cycle. All procedures were carried out in compliance with the guidelines established by the Research Animal Ethics Committee of the Institute of Medicinal Plant Development, Chinese Academy of Medical Sciences (Beijing, China), and in accordance with international standards for animal experiments. The ethics review number is SLXD-20230117028.

### 4.3. Synthesis of PEG-GA@ZIF-8@DOX

ZIF-8@DOX was synthesized using a straightforward one-pot method. Initially, 0.1 g of hexahydrated zinc nitrate was dissolved in 0.4 mL of water, while simultaneously, 20 mg of DOX was dissolved in 2 mL of water. The two solutions were vigorously stirred at 1200 rpm for 5 min. Subsequently, 1 g of 2-methylimidazole was dissolved in 4 mL of water, and it was added slowly, drop by drop, to the previous solution, followed by stirring for 15 min. The resulting red suspension was then subjected to centrifugation and washed three times with alternating ethanol and water. Finally, the sample was dried in a vacuum oven. The synthesis of ZIF-8 followed the same procedure, with the exception of the addition of DOX.

To prepare PEG-GA@ZIF-8@DOX, 50 mg of ZIF-8@DOX was taken and dispersed in 5 mL of deionized water. Subsequently, 50 mg of PEG-GA was added, and the mixture was stirred in a dark environment at room temperature for 48 h. The resulting precipitate was collected through centrifugation and subjected to washing with deionized water before being dried in a vacuum drying oven.

### 4.4. Characterization of Nanoparticles

To characterize the nanoparticles, we dispersed the PEG-GA@ZIF-8@DOX and delicately deposited the droplets onto a carbon film. After drying, we observed the resulting TEM image. We sprinkled the PEG-GA@ZIF-8@DOX powder onto a conductive adhesive, removing any unadhered particles. After coating with a conductive film, we examined the sample under a scanning electron microscope (SEM). We conducted three measurements for each system to determine the size and zeta potential of ZIF-8, ZIF-8@DOX, and PEG-FAZIF-8@DOX using the Nano ZS laser particle size analyzer. The crystal structure of the sample was investigated using the X-ray powder diffraction (XRD) technique, with Cu Kα radiation and a 2θ range of 5–40°. The UV-visible absorption spectra were determined using the Cary100 UV-Vis spectrophotometer from Agilent Technologies (Santa Clara, USA). Infrared spectra were measured using the Nicolet iS 5 infrared spectrometer from Thermo Fisher Scientific (Waltham, MA, USA). Nitrogen adsorption/desorption analysis was conducted using the ASAP 2460 particle analyzer (Micromeritics (Shanghai) Instrument Co., Ltd., Shanghai, China).

### 4.5. Drug Loading and Drug Release

To determine drug loading efficiency (*DLE*), we used a UV-Vis spectrophotometer at an absorption wavelength of 480 nm and established a standard curve of DOX solution. The calculation formula for drug loading efficiency is as follows:

(1) $$DLE(\%)=\frac{w_{1}}{w_{t}}\times 100\%$$

*w*_1_ denotes the weight of the loaded drug in the nanoparticles, and *w_t_* denotes the total weight of the nanoparticles.

The drug’s extracellular release rate was determined using a UV spectrophotometer. PEG-GA@ZIF-8@DOX was dispersed in PBS buffer solutions with different pH values (7.4 and 5.0). This dispersion was placed in dialysis bags with a molecular weight cutoff of 3500 Da. These bags were then placed into release tubes containing the release medium and were immersed in a water bath shaker at 37 °C under gentle agitation at a speed of 100 r/min. Three parallel samples were prepared for each group. At specified time intervals, the supernatant was collected, and fresh PBS was added to maintain the volume. The absorbance was measured within the appropriate range, and the cumulative drug release rate was calculated based on a standard curve.

### 4.6. Cytotoxicity Assay

We assessed cellular toxicity using HepG2 cells with blank nanomaterials, DOX, ZIF-8@DOX, and PEG-GA@ZIF-8@DOX. The HepG2 cells were seeded in a 96-well plate at a density of 1 × 10^4^ cells per well. The plate was then placed in a temperature-controlled cell culture chamber at 37 °C for 24 h. After removing the culture medium, a fresh medium containing various concentrations of PEG-GA@ZIF-8, DOX, ZIF-8@DOX, or PEG-GA@ZIF-8@DOX was added and further incubated for 24 h. After the incubation period, the old medium was replaced with 100 µL of diluted CCK-8 solution. The plate was returned to the incubation chamber, and once the color development was complete, the absorbance was measured at 450 nm using a spectrophotometer. To determine cellular viability, we used the following formula:

Cell viability = [(As − Ab)]/[(Ac − Ab)] × 100%(2)

where As represents the experimental well, Ab signifies the blank well, and Ac denotes the control well.

### 4.7. Cellular Uptake Experiments

The HepG2 cells were placed in 6-well plates with a cell density of 2 × 10^5^ cells per well and cultured for 48 h. Once the cells adhered to the surface, they were gently rinsed with PBS, and the culture medium was replaced with a fresh one containing DOX, ZIF-8@DOX, PEG-GA@ZIF-8@DOX, and PEG-GA + PEG-GA@ZIF-8@DOX, all at a DOX concentration of 6 µg/mL. The control group received fresh culture medium without any drugs. The cells were maintained in a cell culture incubator for an additional 4 h. Subsequently, any remaining drugs were removed by washing with PBS. The cells were then fixed using 4% paraformaldehyde and washed three times with PBS. They were subsequently stained with Hoechst 33342 solution to visualize the cell nuclei. Following three additional PBS washes, fluorescent images were captured using an inverted microscope. DOX exhibited red fluorescence, while Hoechst 33342 emitted blue fluorescence.

### 4.8. Lysosomal Escape

After digesting and centrifuging the HepG2 cells, they were resuspended and placed into 48-well culture plates at a density of 1.5 × 10^4^ cells per well. The plates were then incubated in a cell culture incubator for 48 h to allow them to adhere. After a gentle wash with PBS, the culture medium was replaced with fresh medium containing PEG-GA@ZIF-8@DOX (with a DOX concentration of 6 µg/mL), and the cells were further incubated in the incubator for either 1 h or 4 h. After the specified incubation period, the cells were gently washed with PBS. Subsequently, the cells were stained with Lysotracker Green at a concentration of 75 nM for 20 min, followed by a wash, and 5 min staining with DAPI. After removing the excess dye with PBS, we captured fluorescent images using a fluorescence microscope.

### 4.9. Hemolytic Test

Blood was collected from New Zealand albino rabbits using the cardiac puncture technique. The rabbits were secured in a dorsal position, and the fur around the cardiac region was trimmed. The area was disinfected by gently swabbing it with a 75% alcohol-soaked cotton ball. A sterile syringe was used to perform a vertical puncture at the site where cardiac pulsations were most prominent in order to obtain the blood sample. Fibrinogen was removed from the fresh blood by stirring with a glass rod. The resulting solution was thoroughly washed with physiological saline until the supernatant became colorless. A 2% (*v*/*v*) suspension of rabbit red blood cells was prepared by diluting them with physiological saline. In an EP tube, 1.25 mL of the prepared red blood cell suspension, 1.1 mL of physiological saline, and varying concentrations of the test substance were combined. PBS was used as the negative control, and purified water served as the positive control, with three samples prepared in parallel for each group. After thorough mixing, the sample was promptly incubated in a water bath at 37 ± 0.5 °C. After 3 h, the hemolysis and agglutination reactions were observed, and the absorbance was measured using an ELISA reader. The hemolysis rate was computed using the following formula:

Hemolysis rate (%) = (Asample − Anegative)/(Apositive − Anegative) × 100%(3)

Asample, Anegative, and Apositive, respectively, denote the absorbance values of the sample group, the negative control group, and the positive control group.

### 4.10. Antitumor Research In Vivo

In vivo experiments were conducted using female Balb/c mice that had been injected with H22 cells. The introduction of H22 cells into the peritoneal cavity of the mice led to abdominal swelling and a significant accumulation of ascitic fluid after approximately 5–7 days. Subsequently, the ascitic mice were humanely euthanized through cervical dislocation and immersed in 75% ethanol for a duration of 3 min. The mice were then relocated to a laminar flow hood and placed in a lateral recumbent position. A small incision was made on the abdominal region using scissors, and the skin was gently secured with forceps. The ascitic fluid was carefully drawn into a 15 mL centrifuge tube using a micropipette. A saline solution was employed for washing and subsequent centrifugation, followed by cell counting on a cell counting plate. Subsequently, a cell suspension with a density of 5 × 10^6^ cells/mL was prepared. This suspension was subcutaneously inoculated into the outer side of the right forelimb of each mouse, with a dose of 0.2 mL per mouse. Once the tumor volume reached approximately 100 mm^3^, the mice with tumors were randomly divided into five groups, each comprising eight individuals. Every two days, intravenous injections were administered via the tail vein with either PBS, PEG-GA@ZIF-8 (blank material), DOX, ZIF-8@DOX, or PEG-GA@ZIF-8@DOX solution (DOX: 3 mg/kg) over a duration of 12 days. The body weights were measured, and the tumor volumes were assessed every two days, with the tumor volume calculated using the formula V = L × W^2^/2, where L and W represent the length and width of the tumor, respectively. The relative tumor volume was expressed as V/V_0_, where V_0_ is the initial tumor size of the mouse carrying a tumor. Upon completion of the in vivo efficacy experiment, the mice were euthanized by cervical dislocation, their tumors were dissected and weighed, and the tumor inhibition rate for each treatment group was calculated using the appropriate formula.

Tumor inhibition ratio (%) = (1 − W_t_/W_c_) × 100% (4)

The tumors and major organs (hearts, livers, spleens, lungs, and kidneys) were collected, rinsed with a saline solution, and immersed in tissue fixative for a duration of 24 h. The remaining tissues were enveloped in tin foil and stored in a −80 °C freezer. The fixed organs and tumor tissues were embedded in paraffin, sliced into thin sections, and subjected to hematoxylin and eosin (H&E) staining. The tissue pathology sections were observed and captured using a microscope.

### 4.11. Data Statistics

The data are presented as mean ± SD. Statistical evaluation was performed using two-tailed Student’s *t*-test or one-way ANOVA. “ns” indicates no statistical significance, * indicates *p* < 0.05, ** indicates *p* < 0.01, and *** indicates *p* < 0.001.

## 5. Conclusions

We developed a liver-targeted drug delivery system using ZIF-8 as the carrier, named PEG-GA@ZIF-8@DOX, where PEG-GA is used for surface modification. The GA receptors, which are abundant in liver cells, specifically recognize the GA on PEG-GA@ZIF-8@DOX, enhancing the uptake by these cells. Once taken up by tumor cells, PEG-GA@ZIF-8@DOX demonstrates its ability to escape lysosomes, releasing DOX into the cytoplasm and effectively treating the tumor. The in vivo studies have shown that PEG-GA@ZIF-8@DOX performs significantly better in terms of antitumor effects compared to free DOX and ZIF-8@DOX. Additionally, this system notably reduces weight loss, suggesting its potential to minimize toxicity. However, further investigations are necessary to explore and address the DOX-induced side effects, especially its impact on cardiac toxicity, which will be systematically studied in future research.

## Data Availability

The raw data supporting the conclusions of this article will be made available by the authors, without undue reservation.
